# Preservation of Animal Tissues

**Published:** 1869-01

**Authors:** 


Preservation of Animal Tissues.—Dr. Coolidge showed a foot and lower part of leg injected seven weeks before (48 days). The preparation used was a mixture of carbolic acid, glycerine, sugar and gelatine. The parts were in perfect preservation, soft and natural in appearance. Dr. Coolidge said:

"The carbolic acid and glycerine are the principal preservatives. Last year I assisted at some experiments made at Clermont. The preservative used there for dissections is a solution of the hyposulphite of soda (so I was told). It is not as good as the arseniate of soda, used in the Harvard Medical School. It does not smart if it gets under the nails, as does a solution of arsenic. The arseniate of soda does not either. The solution of the hyposulphite is expensive. Glycerine and sugar was used combined with it, and did tolerably well, but was not permanent. If molasses was substituted for the sugar, it seemed to hasten the decomposition. There was also a smell similar to that produced by the fermentation of molasses. Parts of the muscles of the thigh, injected, I believe, principally with a solution of sugar kept well during the hot weather, hung up without protection, though a mould would appear on it; it remained soft, with no loss of bulk. The most remarkable anatomical preparation, as regards the mere keeping of them, were those of Brissaud and Lakowski. An arm dissected and a heart were in the Musée Orfila, and were a great progress over the usual way. The relations of the parts were preserved ; they were not dried up, as they commonly are.

" The process was a secret, but I was told that it was principally an injection of carbolic acid and sugar, with, perhaps, an after-coat of a solution of gelatine, or gelatine in sugar and water."

